# Metabolic Risk Factors of Alzheimer's Disease, Dementia with Lewy Bodies, and Normal Elderly: A Population-Based Study

**DOI:** 10.1155/2018/8312346

**Published:** 2018-06-03

**Authors:** Chih-Kuang Cheng, Yu-Chien Tsao, Yuan-Chih Su, Fung-Chang Sung, Hsu-Chih Tai, Woon-Man Kung

**Affiliations:** ^1^Stroke Center and Department of Neurology, Linkou Medical Center, Chang Gung Memorial Hospital and College of Medicine, Chang Gung University, Taoyuan, Taiwan; ^2^Department of Internal Medicine, Yonghe Cardinal Tien Hospital, Taipei, Taiwan; ^3^Department of Neurology, Neurological Institute, Taipei Veterans General Hospital, Taipei, Taiwan; ^4^School of Medicine, National Yang-Ming University, Taipei, Taiwan; ^5^Management Office for Health Data, China Medical University Hospital, Taichung, Taiwan; ^6^College of Medicine, China Medical University, Taichung, Taiwan; ^7^Department of Public Health, China Medical University, Taichung, Taiwan; ^8^Department of Exercise and Health Promotion, College of Education, Chinese Culture University, Taipei, Taiwan; ^9^Division of Neurosurgery, Department of Surgery, Taipei Tzu Chi Hospital, Buddhist Tzu Chi Medical Foundation, New Taipei City, Taiwan; ^10^Department of Surgery, School of Medicine, Buddhist Tzu Chi University, Hualien, Taiwan

## Abstract

**Background:**

Alzheimer's disease (AD) and dementia with Lewy bodies (DLB) share many risk factors. Evidence suggests that metabolic risk factors are important to AD; however, their association with DLB is unclear. The risk of cardiovascular diseases (CVD) associated with AD and DLB is also uncertain. Thus, this nationwide, population-based study was designed to evaluate the metabolic and CVD risks in AD and DLB.

**Materials and Methods:**

Data were obtained from the Taiwan National Health Insurance Research Database. AD patients, DLB patients, and normal control (NC) individuals from 1996 to 2013 were enrolled for risk assessment.

**Results:**

In total, 7544 NC individuals, 1324 AD patients, and 562 DLB patients were enrolled. Participants with one or more metabolic risk factors had significantly higher odds of AD or DLB. No significant differences in metabolic risk factors were observed between DLB and AD patients. AD patients had a lower risk of CVD (aHR = 0.67, 95% CI = 0.59–0.76, *p* value < 0.001) and coronary artery disease (CAD) (aHR = 0.59, 95% CI = 0.51–0.69, *p* value < 0.001) than NC. DLB patients had a higher risk of ischemic stroke (aHR = 2.27, 95% CI = 1.68–3.06, *p* value < 0.001) than NC.

**Conclusion:**

Metabolic risk factors are important in AD and DLB. Patients with AD might have a lower risk of CAD and ischemic strokes. Patients with DLB might have a higher risk of ischemic stroke.

## 1. Introduction

Alzheimer's disease (AD) and dementia with Lewy bodies (DLB) are leading causes of dementia [1]. AD is caused by progressive neurodegeneration in the medial temporal lobe, hippocampus formation, and other brain areas with synergistic amyloid and tau proteinopathy [1, 2]. The risk factors for sporadic AD are complex. Apolipoprotein E *ε*4 allele (APOE4) is one of best-known genetic risk factors. Additional risk factors include age, family history of AD, previous stroke, sleep apnea, traumatic brain injury (TBI), and limited education [1]. DLB is characterized by the propagation of aggregated intracellular *α*-synuclein (Lewy bodies) [3]. Risk factors of DLB overlap with that of AD to some extent; for example, APOE4, previous stroke, and TBI are risk factors of both DLB and AD [1, 4–6].

Emerging evidence suggests that metabolic risk factors contribute not only to cardiovascular diseases (CVD) like coronary artery disease (CAD), and ischemic strokes, such as cerebrovascular accident (CVA), but also to dementia [7–9]. Hypertension, diabetes mellitus (DM), and hyperlipidemia are known risk factors for AD [9, 10]. Moreover, a history of CAD or CVA is associated with accelerated cognitive decline in AD patients [8, 11, 12].

However, it is unclear if metabolic risks are also important risk factors of DLB, and it is unclear if AD and DLB patients are more susceptible to CVD. Hence, we conducted a nationwide, population-based, normal control, risk-adjusted, retrospective cohort study in Taiwan to investigate the metabolic risks and the incidence of CVD in patients with AD and DLB. Our purpose is to determine the modifiable metabolic risk factors in DLB and the risk of CVD in AD and DLB to improve the prevention of dementia and CVD.

## 2. Materials and Methods

### 2.1. Study Design

We conducted a retrospective cohort study using health insurance data from the National Health Insurance Research Database (NHIRD), which contained medical information of over 99% of Taiwan citizens. The Longitudinal Health Insurance Database 2000 (LHID2000), a subdataset of the NHIRD, was applied for this study. The LHID2000 contains diagnostic data of the International Classification of Diseases, 9th Revision, Clinical Modification (ICD-9-CM) code, and treatment data from the outpatient visits and hospitalizations from 1996 to 2013 of 1 million random individuals. All participants were encrypted with a dummy number. This study was approved by the Institutional Review Board and the Ethics Committee of China Medical University Hospital, Taiwan (CMUH104-REC2-115 (CR-2)).

**Figure 1:**
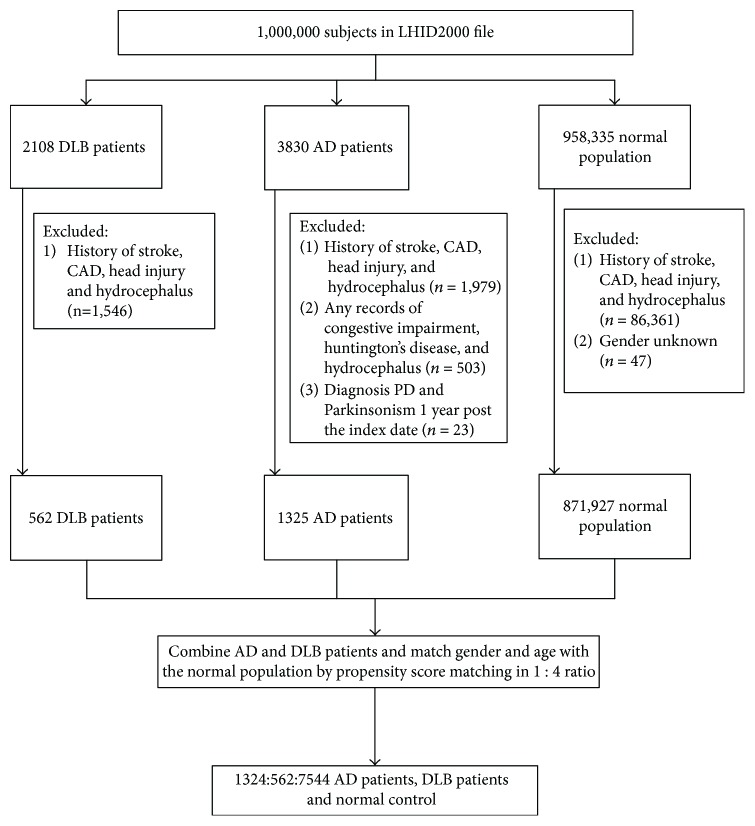
Enrollment flow chart for the study population.

This study investigated AD patients, DLB patients, and normal control (NC) individuals who were enrolled. Both AD and DLB patients were identified using specific criteria. AD patients who met any of the following criteria were included: (1) the ICD-9-CM code 331.0 (AD diagnosis date defined as the index date), or (2) having a diagnosis record of dementia (ICD-9-CM: 290.0–290.4, 294.1, and 331.0–331.2) and receiving acetylcholinesterase inhibitors or memantine treatment (dementia diagnosis date defined as the index date). The exclusion criteria for the AD cohort were patients with (1) any records of cognitive impairment (ICD-9-CM: 331.8, 331.83, and 331.9), Huntington's disease (ICD-9-CM: 333.4), Creutzfeldt-Jakob disease (ICD-9-CM: 046.11 and 046.19), and hydrocephalus (ICD-9-CM: 331.3, 331.4, 331.5, 741.0, and 742.3), (2) a history of stroke (ICD-9-CM: 430–435) or CAD (ICD-9-CM: 410–414) before the index date, and (3) Parkinson's disease (PD) or Parkinsonism diagnosis recorded within 1 year after the index date. For DLB patients, the inclusion criteria were (1) the diagnosis of PD and Parkinsonism (ICD-9-CM: 332, 332.0, 332.1, 333, 333.0, and 333.9) 1 year after diagnosis of dementia (ICD-9-CM: 290.0–290.3, 294.1, and 331.0) (dementia diagnosis date defined as the index date), (2) diagnosis of dementia within 1 year after diagnosis of PD and Parkinsonism (dementia diagnosis date defined as the index date), and (3) ICD-9-CM code 331.82 (DLB diagnosis date defined as the index date). The exclusion criteria for DLB patients included a history of stroke, CAD, head injury (ICD-9-CM: 850–854 and 959.01), or hydrocephalus. Patients with the diagnosis of both AD and DLB were excluded from the study cohorts. The NC population was defined as patients without the diagnosis of AD, DLB, other dementias, or Parkinson's disease. The NC population excluded individuals with a history of stroke or CAD. The study population selection procedure is shown in Figure 1.

### 2.2. Statistical Analysis

The main outcomes of this study were stroke (ICD-9-CM: 430–435) and CAD (ICD-9-CM: 410–414) after the index date. Only inpatient records were used to identify patients with stroke. A history of hypertension (ICD-9-CM: 401–405), diabetes mellitus (ICD-9-CM: 250), and hyperlipidemia (ICD-9-CM: 272) were included as comorbidities. All comorbidities were considered to be significant after at least one inpatient visit or two outpatient visits. In addition, we investigated the use of aspirin, clopidogrel, and warfarin before the index date. Demographic characteristics of sex and age were considered as covariates. We combined the AD and DLB cohorts and matched them with the NC population according to sex and age in a 1 : 4 ratio by propensity score matching. This study compared the risk of the main outcomes, that is, CAD and stroke, among the three cohorts. The risk factors for AD and DLB were also evaluated. The baseline characteristics among the three groups were tested using the chi-square test and ANOVA. Logistic regression was used to evaluate the probable risk factors of AD and DLB. We examined whether the risk of stroke and CAD increases among AD and DLB patients using the Cox proportional hazard regression. A *p* value less than 0.05 was significant. All the statistical analyses were conducted using the statistical software package, SAS, version 9.4 (SAS Institute Inc., Cary, NC).

## 3. Results

The final study population consisted of 7544 NC individuals, 1324 AD patients, and 562 DLB patients (Table 1). The number of female participants was higher than male participants in the AD cohort. Contrarily, the DLB cohort included a higher number of male participants. The difference in the average ages among the three cohorts was insignificant (*p* value = 0.26). Participants under 65 years were less than 20% in each cohort. The distributions of baseline comorbidities differed among the NC, AD, and DLB groups (*p* value < 0.001). The medication status was also significantly different (*p* value < 0.01) among the cohorts. The proportions of diabetes mellitus and hyperlipidemia were higher in AD patients than in DLB patients. There were 58.4% and 60.5% hypertensive individuals in the AD group and the DLB group, respectively.

Between AD and NC, compared to participants without any metabolic risk factors, the participants with one or more metabolic risk factors had significantly higher odds of AD (Table 2). The odds ratio of AD was 4.9 (95% CI = 4.01–5.99, *p* value < 0.001) in patients with three metabolic risk factors. For DLB and NC, the logistic regression showed an odds ratio of 3.64 for DLB (95% CI = 2.65–5, *p* value < 0.001) in patients with three metabolic risk factors. Between DLB and AD, there were no significant odds of DLB in patients with any metabolic risk factors. The male patients had low odds of AD (OR = 0.82, 95% CI = 0.72–0.92, *p* value < 0.01), but high odds of DLB (OR = 1.42, 95% CI = 1.19–1.69, *p* value < 0.001).

The results of the Cox model indicated that the AD patients had a lower risk of outcomes (aHR = 0.67, 95% CI = 0.59–0.76, *p* value < 0.001) than the NC population (Table 3). Separated, the risks of CAD and stroke were 0.59-fold (95% CI = 0.51–0.69, *p* value < 0.001) and 1.02-fold (95% CI = 0.8–1.3, *p* value > 0.05), respectively, in AD patients. Compared with the NC cohort, there was a higher stroke risk in the DLB group (aHR = 2.08, 95% CI = 1.58–2.73, *p* value < 0.001). The risk of both outcomes combined was also higher (aHR = 1.23, 95% CI = 1.06–1.44, *p* value < 0.01). However, the risk of CAD alone was negligible (aHR = 1.04, 95% CI = 0.86–1.24, *p* value > 0.05) among DLB patients. The risk of outcomes in males was higher than that in females (aHR = 1.14, 95% CI = 1.06–1.23, *p* value < 0.001). The risk of outcomes increased 1.03-fold per year with increasing age (95% CI = 1.03–1.04, *p* value < 0.001). The aHR outcomes of hypertension and diabetes mellitus were 1.39 (95% CI = 1.28–1.51, *p* value < 0.001) and 1.2 (95% CI = 1.09–1.34, *p* value < 0.001), respectively. Hyperlipidemia reduced the risk of stroke (aHR = 0.7, 95% CI = 0.55–0.9, *p* value < 0.01), but did not affect the risk of outcomes (aHR = 0.9, 95% CI = 0.81–1, *p* value > 0.05) and CAD (aHR = 0.96, 95% CI = 0.86–1.09, *p* value > 0.05). There was no significant effect on the risk of outcomes with aspirin (aHR = 0.98, 95% CI = 0.89–1.09, *p* value > 0.05), clopidogrel (aHR = 1.25, 95% CI = 0.72–2.16, *p* value > 0.05), and warfarin (aHR = 0.66, 95% CI = 0.38–1.13, *p* value > 0.05). Additionally, we divided stroke outcome into ischemic and hemorrhagic stroke. The ischemic stroke risk was insignificant among AD patients (aHR = 1.01, 95% CI = 0.77–1.33, *p* value > 0.05) when compared to the NC population (Table 4). In contrast, the DLB group had a higher ischemic stroke risk (aHR = 2.27, 95% CI = 1.68–3.06, *p* value < 0.001). The aHRs of hemorrhagic stroke among AD and DLB patients were insignificant (for AD: aHR = 1.04, 95% CI = 0.61–1.77, *p* value > 0.05; for DLB: aHR = 1.4, 95% CI = 0.7–2.8, *p* value > 0.05).

## 4. Discussion

In this retrospective cohort study, female patients were more likely to have AD, while male patients were more likely to have DLB. Patients with any one of the metabolic risk factors considered, had an increased risk of having AD or DLB. Patients who had three comorbid risk factors (hypertension, diabetes mellitus, and hyperlipidemia) simultaneously were at higher risk of having AD or DLB than patients with a single risk factor. The consequences of metabolic risk factors for AD are similar to those for DLB. Patients with AD had a lower risk, while patients with DLB had a higher risk of all CVD outcomes than the NC population. When outcomes were separated, patients with AD had a lower risk of CAD, and patients with DLB had a higher risk of ischemic stroke than the NC population. Patients with AD or DLB had no increased risk of hemorrhagic stroke.

We found that AD was more prevalent among female patients, while DLB was more prevalent among male patients. Sex differences between AD and DLB have been reported [13]. The age at onset of AD may be lower in women than in men with the same copies of the APOE *ε*4 allele [14]. Male sex is a known risk factor for DLB [3, 5]. The cause of these sex differences remains uncertain and need to be clarified.

We found that hypertension significantly increased the risk of both AD (OR = 2.29, 95% CI = 1.95–2.69) and DLB (OR = 2.74, 95% CI = 2.20–3.42). Hypertension is a risk factor for dementia [9]. Studies have shown that hypertension increases the risk of AD, and that antihypertensive treatment can reduce the risk of AD [10]. The relationship between hypertension and DLB is less understood. In a neuropathological study, patients with DLB pathology were found to have a significantly higher incidence of hypertension [15].

We also found that diabetes mellitus increased the risk of AD (OR = 2.28, 95% CI = 1.64–3.18) and DLB (OR = 2.23, 95% CI = 1.38–3.62). Diabetes mellitus is a risk factor for cognitive decline [10]. Insulin insufficiency increases the risk of AD [10]. Either an excessive glucose level or an insulin dysfunction may play a role in AD [10]. There is limited information about the effect of diabetes on DLB. Although diabetes was a risk factor for DLB in our study, it did not alter the risk of DLB in another study [5]. This conflicting result may have arisen because of the different diagnostic criteria for diabetes in different populations, and further studies are warranted to clarify if diabetes causes DLB.

Hyperlipidemia was another risk factor that increased AD (OR = 3.70, 95% CI = 2.78–4.93) and DLB (OR = 2.66, 95% CI = 1.65–4.29). Dysfunction of lipid metabolism plays an important role in dementia. High total serum cholesterol levels are associated with dementia and AD [10]. APOE is an important class of lipoprotein in lipid metabolism, and APOE4, an allele of APOE, is implicated in AD. APOE4 is also more frequent in DLB patients than in controls [5]. Nevertheless, the mechanism by which lipid disorders affect DLB is obscure. Considering its close association with dementia and that it is a modifiable risk factor, we suggest that studies focus more on lipid disorders in future DLB studies.

Having three metabolic risk factors simultaneously further increased the risk of AD (OR = 4.90, 95% CI = 4.01–5.99) and DLB (OR = 3.64, 95% CI = 2.65–5.00). The hazard of metabolic risk factors is similar in AD and DLB. AD and DLB share many similar risk factors such as depression, smoking, and the APOE *ε*4 allele [5]. Cerebrovascular lesions are also frequent in both AD and DLB [16, 17]. AD and DLB show clinical and pathological similarity, and metabolic risk factors may have similar roles in the risk of developing AD and DLB. Summarizing, better lifestyle modifications and medication for the treatment of metabolic risk factors may not only prevent stroke and CAD but also AD and DLB.

In a risk-adjusted long-term follow-up, we found that AD had a decreasing risk of CAD (aHR = 0.59, 95% CI = 0.51–0.69), while DLB had an increasing risk of ischemic stroke (aHR = 2.27, 95% CI = 1.68–3.06). AD is associated with atherosclerosis [18]. Evidence has shown that cerebral atherosclerosis, involving extracranial or intracranial arteries, contributes to the cognitive decline in AD [19, 20]. Coronary atherosclerosis also worsens cognitive function in AD [11]. These facts reflect the hemodynamic compromise or hypoperfusion of the brain which correlates with AD deterioration [21–23]. However, AD itself does not necessarily increase the risk of CAD. In fact, a recent study showed that APOE *ε*4 does not interact with CAD [24]. Interestingly, our results implied that patients with AD might have protective factors to CAD, with the underdiagnosis of CAD or ischemic stroke in AD having been one possible explanation. However, we did not find a similar effect in DLB, making it less likely. A change in eating behaviour in AD was another possible explanation. Anorexia and weight loss, which are common in advanced AD [25], may reduce blood sugar or lipid level and decrease the risk of atherosclerosis. A change in amyloid *β* (A*β*) metabolism may affect the risk of CAD in AD. Extracellular deposition of A*β* in the brain is the pathological hallmark of AD [26]. A dysfunction of A*β* production and clearance in the central nervous system may contribute to the accumulation of cerebral A*β* in late onset AD [27, 28]. Higher plasma A*β* also correlates with cerebral white matter lesions and ischemic heart disease [29]. In short, the change in the A*β* metabolic pathway in AD may be associated with cerebrovascular and cardiovascular risks, and further investigations are warranted.

A history of stroke is more frequently observed in patients with DLB [5]. White matter hyperintensities on cerebral T2-weighted magnetic resonance imaging are higher in DLB than in AD patients [17]. DLB usually shows hypoperfusion in the striatum and the visual cortex [3]. All these studies suggest that DLB correlates with the function of cerebral small vessels. Postural hypotension is commonly seen in DLB [3], and is also a potential risk factor for ischemic stroke. Intracellular *α*-synuclein aggregation is characteristic of DLB [1]. Studies also show a potential role for *α*-synuclein in ischemic stroke modulation [30]. Further studies are required to determine the mechanism of stroke in DLB.

Cerebral microbleeds are small hemosiderin deposits in the brain and are not unusual in AD or DLB [31, 32]. The gradual spreading of the intracellular aggregation of hyperphosphorylated tau (P-tau) in the brain is associated with AD progression [1]. Studies also reveal the association between cerebral microbleeds and P-tau [33, 34]. Interestingly, our data did not reveal increased hemorrhagic stroke in both AD and DLB patients when compared to NC individuals. This may imply that the pathological processes for intracerebral hemorrhage and cerebral microbleeds may differ in AD and DLB.

Limitations were inevitable in this study. First, subjects were classified based on the main clinical diagnosis and bias from misclassification was possible. Dementia is a complex syndrome and accurate diagnosis is challenging because of the overlapping clinical presentation. Although we used multiple methods during the inclusion process to identify the diagnosis and to minimize misclassification, a few atypical cases may still present difficulties in classification. Second, the severity of metabolic risk factors and their different treatment methods can potentially affect the risk of CAD or stroke. Third, this was a Taiwan-based study, and the influence of risk may vary among different racial groups. Fourth, this was a retrospective study, and selection and information biases cannot be completely excluded.

## 5. Conclusions

Hypertension, diabetes mellitus, and hyperlipidemia are risk factors of AD and DLB. Multiple metabolic risk factors, when simultaneously present, might aggravate the risk of AD and DLB. The control of metabolic risk factors is crucial for the prevention of AD and DLB. Patients with AD might have a lower risk of CAD, while patients with DLB might have a higher risk of ischemic stroke. Our study suggests that better lifestyle modifications and medications for the treatment of metabolic risk factors may lower the risk of AD and DLB. Furthermore, stroke prevention is important in patients with DLB. Further studies to understand the mechanism of stroke in DLB are warranted.

## Figures and Tables

**Table 1:** Demographic and background characteristics of NC, AD, and DLB patients.

	NC	AD	DLB	*p* value
*N*	7544	1324	562	
Sex, f/m	3758/3786	768/556	242/320	<0.001
Age				
<50	216 (2.9)	39 (2.9)	15 (2.7)	
50–64	939 (12.4)	188 (14.2)	50 (8.90)	
≥65	6389 (84.7)	1097 (82.9)	497 (88.4)	
Mean (SD)	74.5 (11.1)	74.2 (11.4)	75.1 (10.3)	0.26^∗^
Comorbidity				
Hypertension	2777 (36.8)	773 (58.4)	340 (60.5)	<0.001
Diabetes mellitus	1132 (15.0)	387 (29.2)	142 (25.3)	<0.001
Hyperlipidemia	1201 (15.9)	466 (35.2)	146 (26.0)	<0.001
Drug				
Aspirin	1139 (15.1)	468 (35.3)	186 (33.1)	<0.001
Clopidogrel	21 (0.28)	18 (1.36)	7 (1.25)	<0.001
Warfarin	45 (0.6)	20 (1.51)	5 (0.89)	0.002

Chi-square test; ^∗^ANOVA. NC, normal control; AD, Alzheimer's disease; DLB, dementia with Lewy bodies; SD, standard deviation.

**Table 2:** Risk factors for AD and DLB patients.

Risk factors	Multivariable ORs (95% CI)		
	AD versus NC	DLB versus NC	DLB versus AD
Sex			
Female	1 (reference)	1 (reference)	1 (reference)
Male	0.82 (0.72–0.92)^∗∗^	1.42 (1.19–1.69)^∗∗∗^	1.75 (1.43–2.15)^∗∗∗^
Age, per year	1.0 (0.99–1.0)	1.0 (0.99–1.01)	1 (0.99–1.01)
Combination of metabolic risk factors (hypertension, diabetes, and hyperlipidemia)			
(− − −)	1 (reference)	1 (reference)	1 (reference)
(+ − −)	2.29 (1.95–2.69)^∗∗∗^	2.74 (2.2–3.42)^∗∗∗^	1.16 (0.89–1.52)
(− + −)	2.28 (1.64–3.18)^∗∗∗^	2.23 (1.38–3.62)^∗∗∗^	0.91 (0.52–1.6)
(− − +)	3.7 (2.78–4.93)^∗∗∗^	2.66 (1.65–4.29)^∗∗∗^	0.66 (0.39–1.11)
(+ + −)	3.08 (2.42–3.92)^∗∗∗^	3.6 (2.6–4.99)^∗∗∗^	1.18 (0.81–1.73)
(+ − +)	3.63 (2.94–4.49)^∗∗∗^	3.25 (2.37–4.46)^∗∗∗^	0.88 (0.62–1.26)
(− + +)	3.58 (2.48–5.17)^∗∗∗^	2.14 (1.1–4.17)^∗^	0.62 (0.3–1.28)
(+ + +)	4.9 (4.01–5.99)^∗∗∗^	3.64 (2.65–5)^∗∗∗^	0.7 (0.5–0.99)

+, presence; −, absence; CI, confidential interval; OR, odds ratio; NC, normal control; AD, Alzheimer's disease; DLB, dementia with Lewy bodies. Adjusted sex and age in logistic regression. ^∗^*p* < 0.05, ^∗∗^*p* < 0.01, and ^∗∗∗^*p* < 0.001.

**Table 3:** Multivariable analysis of risk of CAD and stroke in Cox proportional hazard regression.

Risk factors	All outcomes	CAD	Stroke
	Adjusted HR (95% CI)	Adjusted HR (95% CI)	Adjusted HR (95% CI)
NC	1 (reference)	1 (reference)	1 (reference)
AD	0.67 (0.59–0.76)^∗∗∗^	0.59 (0.51–0.69)^∗∗∗^	1.02 (0.8–1.3)
DLB	1.23 (1.06–1.44)^∗∗^	1.04 (0.86–1.24)	2.08 (1.58–2.73)^∗∗∗^
Sex			
Female	1 (reference)	1 (reference)	1 (reference)
Male	1.14 (1.06–1.23)^∗∗∗^	1.11 (1.02–1.21)^∗^	1.26 (1.07–1.49)^∗∗^
Age, per year	1.03 (1.03–1.04)^∗∗∗^	1.03 (1.02–1.03)^∗∗∗^	1.05 (1.04–1.06)^∗∗∗^
Hypertension (yes versus no)	1.39 (1.28–1.51)^∗∗∗^	1.39 (1.27–1.53)^∗∗∗^	1.37 (1.14–1.64)^∗∗∗^
Diabetes mellitus (yes versus no)	1.2 (1.09–1.34)^∗∗∗^	1.15 (1.02–1.29)^∗^	1.43 (1.15–1.78)^∗∗^
Hyperlipidemia (yes versus no)	0.9 (0.81–1)	0.96 (0.86–1.09)	0.7 (0.55–0.9)^∗∗^
Aspirin (yes versus no)	0.98 (0.89–1.09)	0.95 (0.85–1.07)	1.1 (0.89–1.37)
Clopidogrel (yes versus no)	1.25 (0.72–2.16)	1.29 (0.69–2.41)	1.14 (0.36–3.58)
Warfarin (yes versus no)	0.66 (0.38–1.13)	0.6 (0.31–1.15)	0.84 (0.31–2.27)

HR, hazard ratio; CI, confidence interval; NC, normal control; AD, Alzheimer's disease; DLB, dementia with Lewy bodies. Adjusted sex, age, hypertension, diabetes mellitus, hyperlipidemia, aspirin, clopidogrel, and warfarin in Cox proportional hazards regression. ^∗^*p* < 0.05, ^∗∗^*p* < 0.01, and ^∗∗∗^*p* < 0.001.

**Table 4:** Multivariable analysis of the risk of ischemic stroke and hemorrhagic stroke in Cox proportional hazard regression.

Risk factors	Ischemic stroke	Hemorrhage stroke
	Adjusted HR (95% CI)	Adjusted HR (95% CI)
NC	1 (reference)	1 (reference)
AD	1.01 (0.77–1.33)	1.04 (0.61–1.77)
DLB	2.27 (1.68–3.06)^∗∗∗^	1.4 (0.7–2.8)
Sex		
Female	1 (reference)	1 (reference)
Male	1.16 (0.97–1.4)	1.74 (1.21–2.49)^∗∗^
Age, per year	1.05 (1.04–1.06)^∗∗∗^	1.05 (1.03–1.07)^∗∗∗^
Hypertension (yes versus no)	1.34 (1.09–1.64)^∗∗^	1.5 (1.03–2.19)^∗^
Diabetes mellitus (yes versus no)	1.48 (1.16–1.88)^∗∗^	1.25 (0.76–2.06)
Hyperlipidemia (yes versus no)	0.75 (0.57–0.98)^∗^	0.52 (0.29–0.95)^∗^
Aspirin (yes versus no)	1.21 (0.95–1.53)	0.76 (0.46–1.27)
Clopidogrel (yes versus no)	0.91 (0.22–3.68)	2.36 (0.32–17.36)
Warfarin (yes versus no)	1.05 (0.39–2.82)	

HR, hazard ratio; CI, confidence interval; NC, normal control; AD, Alzheimer's disease; DLB, dementia with Lewy bodies. Adjusted sex, age, hypertension, diabetes mellitus, hyperlipidemia, aspirin, clopidogrel, and warfarin in Cox proportional hazards regression. ^∗^*p* < 0.05, ^∗∗^*p* < 0.01, and ^∗∗∗^*p* < 0.001.

## Data Availability

The data used to support the findings of this study are available from the corresponding author upon request.
